# Revision of the genus *Eadmuna* Schaus, 1928 (Lepidoptera, Mimallonidae) with a description of a new species from French Guiana

**DOI:** 10.3897/zookeys.494.9208

**Published:** 2015-04-06

**Authors:** Ryan A. St. Laurent, Jason J. Dombroskie

**Affiliations:** 1 Cornell University, Comstock Hall, Department of Entomology, Ithaca, NY 14853-2601 USA

**Keywords:** Brazilian Atlantic forest, *Cicinnus*, *Eadmuna guianensis*, Guyana

## Abstract

The genus *Eadmuna* Schaus, 1928 is revised to include four species. *Eadmuna guianensis* **sp. n.**, is described from French Guiana and Guyana. The holotype of *Perophora pulverula* Schaus, 1896, currently placed in *Cicinnus* Blanchard, 1852, is determined to be a previously unrecognized female *Eadmuna*, and is transferred accordingly as *Eadmuna pulverula* **comb. n..** *Eadmuna paloa* Schaus, 1933, **rev. status**, is removed from synonymy with the type species *Eadmuna esperans* (Schaus, 1905). *Eadmuna esperans*, *Eadmuna paloa*, and *Eadmuna pulverula* may be of conservation concern due to their limited extent of occurrence and endemicity to the highly imperiled Brazilian Atlantic forest.

## Introduction

The strictly New World and primarily Neotropical Mimallonoidea, comprised of the sole family Mimallonidae, presently consists of barely more than 200 described species in 28 genera (Herbin 2012). Phylogenetic relationships within the family Mimallonidae are not well understood, nor has there been a modern thorough treatment of the family (Herbin 2012); Schaus (1928) was the last to treat the family as a whole. The two subfamilies proposed by Schaus, the Mimalloninae and the Lacosominae, were determined to be inadequately supported by both Pearson (1951, 1984) and Franclemont (1973) based on the fact that the trait originally used to separate the subfamilies (presence/absence of frenulum) was inconsistent, and the presence of the frenulum was deemed plesiomorphic by Herbin (2012). However, the subfamilies were maintained by Becker (1996). Until a sufficient monograph or generic revision of the family is completed, the relationships within the family will remain poorly understood; therefore, the subfamily classification used here follows Herbin (2012), without any currently recognized subfamilies.

Various genera in Mimallonidae lack well-defined unifying characters. *Cicinnus* Blanchard, 1852 and *Psychocampa* Grote & Robinson, 1866 in particular have been used by authors, essentially from Schaus until present, including Herbin (2012) and Herbin and Mielke (2014), to place newly described species of Mimallonidae without attempting to delineate synapomorphies for these genera. Additionally, the majority of genera in the family are small and frequently monotypic, half of the 28 currently recognized genera have fewer than three species. This is not surprising considering the variation in external wing morphology within the family, which was used by Schaus (1928) as the primary means for generic classification. Schaus (1928) provided a key to genera after separating them into the aforementioned subfamilies. The key relies primarily on venation and wing morphology traits, some of which are highly superficial and variable and are difficult to apply consistently in diagnoses, such as the appearance of the tornus. The basis of the subfamilies in the key, namely the presence or absence of the frenulum, was incorrectly reported by Schaus for a number of genera, thus the key lacks reliability. In addition to the issues presented by Schaus’ key, Mimallonidae are frequently sexually dimorphic and this has resulted in the description of conspecific sexes as different species.

Currently the genus *Eadmuna* Schaus, 1928, contains a single species, *Eadmuna esperans* (Schaus, 1905). An additional species, *Eadmuna paloa* Schaus, 1933, was treated as a synonym of *Eadmuna esperans* by Becker (1996). The present work aims to determine the validity of names currently assigned to *Eadmuna* and a species assigned to the genus *Cicinnus*. Additionally, we will establish genus specific synapomorphies of *Eadmuna*, providing adequate support for placing a new species from French Guiana and Guyana within the genus.

## Methods

Dissections were performed as in Lafontaine (1987), however, genitalia slides were not created in order to allow for three-dimensional analysis. Genitalia and abdomens are preserved in glycerol in microvials. Morphological, including genitalic, terminology follows Lemaire and Minet (1999). Wing venation terminology follows Franclemont (1973).

The holotypes of *Eadmuna paloa* and *Perophora pulverula* Schaus, 1896 were dissected and at least one specimen from each locality was dissected. In some cases only one specimen from a given locality was available for study. The genitalia slide of the holotype of *Eadmuna esperans* was unavailable; however, topotypical *Eadmuna esperans* were dissected. All known *Eadmuna* specimens from the following institutions were examined:

AMNH American Museum of Natural History, New York, New York, USA

CMNH Carnegie Museum of Natural History, Pittsburgh, Pennsylvania, USA

CNC Canadian National Collection of Insects, Arachnids and Nematodes, Ottawa, Ontario, Canada.

CUIC Cornell University Insect Collection, Ithaca, New York, USA

FSCA Florida State Collection of Arthropods, Gainesville, Florida, USA

MGCL McGuire Center for Lepidoptera & Biodiversity, Gainesville, Florida, USA

USNM National Museum of Natural History [formerly United States National Museum], Washington D.C., USA

Figures were manipulated with Adobe Photoshop CS4 (Adobe 2008). Images of adults were edited so that the best “half” is figured, and mirror images of the best half are figured so that the left half is shown for each specimen.

Maps were created with SimpleMappr (Shorthouse 2010) and edited with CS4. All geographical co-ordinates are approximate, and are based on the localities provided on specimen labels. GPS data was acquired with Google Earth.

## Results

### Eadmuna

Taxon classificationAnimaliaLepidopteraMimallonidae

Schaus, 1928

#### Type species.

*Cicinnus esperans* Schaus, 1905

#### Diagnosis.

*Eadmuna* can be recognized by broad wings and silvery-gray ground color accented by varying degrees of brown. The forewing bears a discal cell as a hyaline or sub-hyaline patch bisected by the M_2_ vein creating two separate windows. The hindwings lack any such hyaline markings. Although this marking is not unique within Mimallonidae, this character combined with the following two additional characters: the absence of any straight, continuous, vertical or diagonal postmedial lines and the presence of smooth wing margins; are diagnostic for the genus. Male genitalia are simple with a pointed, teardrop-shaped uncus, broad, ovoid tegumen with a pair of prominent, subtriangular, ridged lobes ventrally.

#### Description.

**Male.** *Head*: Very small, scales on frons swept forward, eyes large comprising roughly two-thirds of head area, bordered posteriorly by darkbrown scales, border of darker scales continues down head reaching beneath labial palpi, labial palpi very small, segments smaller distally, hardly extending beyond frons, basal two segments tufted ventrally, dorsally covered in darkbrown scales greatly contrasting with overall straw coloration of head. Antenna bipectinate, scape and pedicel tufted. Ocelli and putative chaetosemata present. *Thorax*: Densely covered in long, hair-like scales interspersed with widened, darker, petiolate scales giving a speckled appearance. *Legs*: Vestiture thick, scales long, especially on femur and tibia, coloration as for thorax, petiolate scales present. Tibial spurs about one fifth length of tibia, thick, triangular in cross section, ridged, ridges finely serrate along ventral length. *Forewing dorsum*: Forewing length: 16–20 mm, n=40. Triangular, convex outer margins becoming concave near apex in some species, apex accentuated. Silvery gray-brown ground color with extensive speckling due to dark, petiolate scales in similar manner to that of thorax. Discal spot prominent, hyaline or partially covered in translucent scales, with M_2_ vein covered in dark scales separating hyaline patch into two distinct regions. Postmedial line usually present, though often faint, bulged in costal half, brown, dentate. Overall, scales become smaller and finer distally from wing base. *Forewing venter*: As for dorsum but usually lighter, postmedial lines generally more pronounced. *Hindwing dorsum*: Rounded, somewhat accentuated anal angle, essentially bearing same coloration and scale pattern as forewings though postmedial line usually fainter, if present. No hyaline patches present. Spatulate scales denser on inner margin. *Hindwing venter*: As for dorsum but usually lighter, postmedial lines generally more pronounced, frenulum with single bristle. *Wing venation*: As for *Cicinnus melsheimeri* (Harris, 1841) but R_4_ + R_5_ much shorter stalked. *Abdomen*: Somewhat compressed laterally, short, depth equal to that of thorax, rather triangular due to sudden truncation to slightly upturned tip, coloration a continuation of thoracic color, matching essentially dorsal wing coloration. *Genitalia*: Simple, uncus abruptly narrowed at base, extended apically. Tegumen broad, ovoid, with prominent, subtriangular, ridged lobes. Anal tube barely discernable, lightly sclerotized, with apex roughly halfway to distal tip of uncus. Valves simple, lightly sclerotized, basal half wider than distal half, sacculus half to one third width of valve at base, extending to half or two-thirds valve length. Juxta ventrally with quadrate lip and with two triangular arm-like spurs, one on either side of phallus. Juxtal spurs reach roughly midway along length of phallus. A small relatively quadrate sclerotized plate present dorsally to juxta/phallus. Vinculum broadly ovoid though flattened on dorsal and ventral margins, somewhat quadrate. Phallus simple, cylindrical, vesica sac-like or elongated with scobinate patch or with multidentate cornutus. **Female.** Similar to male except for: *Head*: Eyes greater than two thirds area of head, labial palpi smaller, region of brown scales bordering posterior of eyes thicker, extending to prothorax ventrally. *Legs*: Small scales nearly completely cover tibial spurs. *Forewing dorsum*: Forewing length range: 22–24 mm, n=3. Compared to male, forewing much broader overall, postmedial region lighter, more silvery gray than medial area, hyaline discal spot large, prominent. Postmedial line present, more pronounced than for male, brown, dentate, narrowly interrupted by veins, dark wedge where postmedial line meets costa. Antemedial lines present, bilobed, B-shaped. *Forewing venter*: As for dorsum, but lighter, postmedial line more contrasting. *Hindwing dorsum*: Broader, hardly accentuated anal angle, essentially bearing same coloration as forewings. Unlike males, entire hindwing, save for postmedial line, concolorous silvery gray, without a brown edge and without darker medial area present in forewings. Dentate postmedial line dark and well pronounced, narrowly interrupted by veins, slightly darker than that of forewing. *Hindwing venter*: As for dorsum, but lighter, postmedial line more contrasting, frenulum rudimentary with numerous bristles hidden by hindwing scales. *Wing venation*: As for male but Rs appears to originate closer to middle of cell. *Abdomen*: Much broader than that of male. Coloration a continuation of thoracic color, though darkening somewhat distally. Two very elongated sclerotized plates present on venter of eighth segment. *Genitalia*: Papillae anales elongated or stocky, covered in fine setae, apophyses posteriores shorter or same length as apophyses anteriores. Ductus bursae short, ostium opening immediately into corpus bursae. Corpus bursae, round, with or without sclerotized structures reinforcing membrane, elongated appendix bursae.

#### Remarks.

Despite Schaus’ (1928) comment that *Eadmuna* genitalia are allied to those of *Psychocampa* (unspecified species), this has not been found when comparing *Eadmuna* genitalia to those of some representative *Psychocampa*. No *Eadmuna* genitalia resemble any of the *Psychocampa* genitalia figured in Herbin (2012), including *Psychocampa kohlii* Herbin which greatly resemble the genitalia of the type species *Psychocampa concolor* Grote & Robinson, 1866 (Herbin 2012).

*Aceclosteria villaricensis* (Schaus, 1933) was originally described in *Eadmuna*. Currently the genus *Aceclosteria* Vuillot, 1893 contains one species, *Aceclosteria mus* Vuillot, 1893. Previously Schaus described a female *Aceclosteria* specimen as *Eadmuna villaricensis* due to it being allied with “*Eadmuna esperanza*,” [sic] (Schaus, 1933) though the two species are quite dissimilar. For instance, *Aceclosteria mus* has a continuous, non-dentate postmedial line. Additionally, in a single male genitalia dissection of *Aceclosteria mus* from Rio Grande do Sul, Brazil (CUIC genitalia dissection 10-8-14:2), the genitalia were found to be highly complex structurally and asymmetrical, completely unlike *Eadmuna*. Becker (1996) synonymized *Eadmuna villaricensis* with *Aceclosteria mus*. An external examination of the holotype of *Eadmuna villaricensis* supports Becker’s synonymy.

One or two other undescribed species from Costa Rica are currently considered to belong to *Eadmuna* by Daniel Herbin (pers. comm.). These golden-colored species, superficially somewhat similar to *Eadmuna guianensis*, new species and *Eadmuna esperans*, have broad wings, dentate postmedial lines, and bisected forewing hyaline areas. However, the genitalia are very distinct (MGCL dissection number 9-24-14:1). In one of these undescribed species, the uncus is not truncate and is rather triangular and flattened apically, the juxta has two extremely long, curved tusk-like projections, pointed outwards above the phallus. Finally, somewhat triangular tegumen lobes are present, as in *Eadmuna*, but are significantly elongated and without numerous ridges as in *Eadmuna*. Thus, these species from Costa Rica cannot be considered *Eadmuna*.

The geographic distribution of the species *Eadmuna esperans* and *Eadmuna paloa*, and possibly *Eadmuna pulverula*, very clearly follows the Atlantic coastal rainforest of Brazil (see Figure 18) (IBGE). This biome is of particular conservation interest due to a massive loss of habitat, such that it has been estimated that only approximately 11% of the Brazilian Atlantic forest remains (Ribeiro et al. 2009). The association of these two or three species with this biome, along with the almost complete lack of recent material of these species in any of the visited collections, presents further justification for the conservation of this area of high species richness (Ribeiro et al. 2009).

#### Key to species of Eadmuna*

**Table:** 

1	Antemedial and postmedial lines weakly defined, usually only postmedial line visible (Figs 1–6) (male)	**2**
–	Antemedial and postmedial lines well defined (Figs 7, 8) (female)	**4**
2	Silvery-gray forewing elongated relative to hindwing (Figs 5, 6); with large hyaline areas, devoid of covering of scales, male vesica with a large straight cornutus that is fused to progressively smaller, parallel cornuti (Fig. 14)	***Eadmuna paloa* (part)**
–	Silvery-brown to brown forewing not particularly elongated relative to hindwing (Figs 1–4); weakly to moderately falcate with small yellowish opaque hyaline discal markings, cornutus if present, not straight (Fig. 12)	**3**
3	Wing silvery-brown, weakly falcate (Figs 1, 2), vesica with scobinate patch (Fig. 13), occurring in southern and southeastern Brazil	***Eadmuna esperans***
–	Wing darker brown, more falcate (Figs 3, 4), vesica with curved, spiked cornutus (Fig. 12), occurring in northern South America (Guyana and French Guiana)	***Eadmuna guianensis* sp. n.**
4	Forewing apex rounded, large hyaline discal mark (Fig. 7), corpus bursae firm, round, with heavily-sclerotized, internal bar-like structures reinforcing membrane (Figs 15, 16), venter of abdomen devoid of markings	***Eadmuna paloa* (part)**
–	Forewing slightly falcate with smaller discal mark (Fig. 8), corpus bursae small, bag-like, without signum or cornuti (Fig. 17), dark longitudinal line on venter of abdomen	***Eadmuna pulverula***

* Note: the male of *Eadmuna pulverula* and the females of *Eadmuna esperans* and *Eadmuna guianensis* are unknown

**Figures 1–8.**
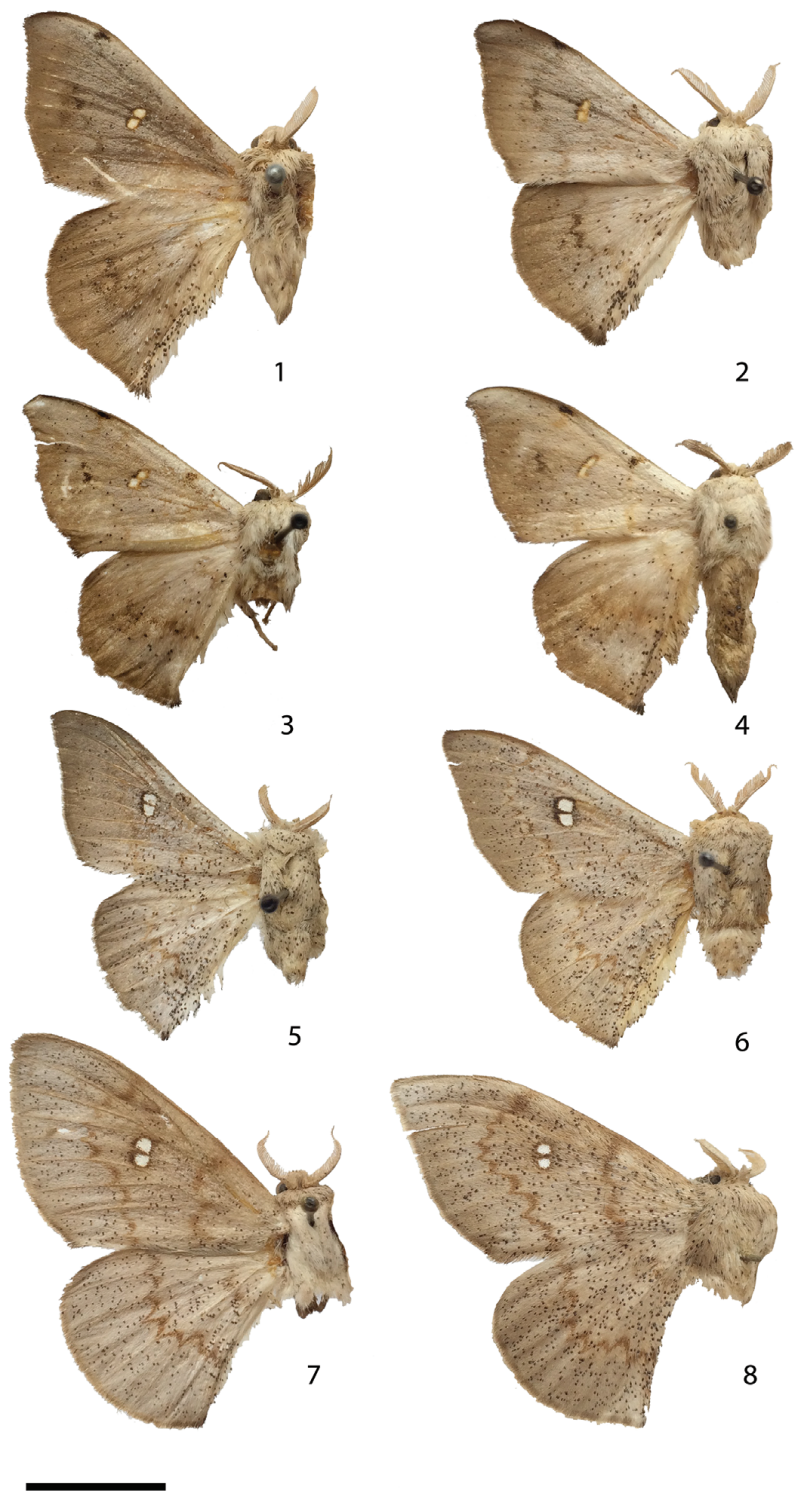
*Eadmuna* adults. **1** *Eadmuna esperans* holotype male, Espírito Santo, Brazil (image inverted laterally) [USNM] **2** *Eadmuna esperans* male, Jaraguá [do Sul], Santa Catarina, Brazil [CUIC] **3** *Eadmuna guianensis* holotype male, Mana River, French Guiana [CMNH] **4** *Eadmuna guianensis* paratype male, Mana River, French Guiana [CMNH] **5** *Eadmuna paloa* holotype male, São Paulo, Brazil (image inverted laterally) [USNM] **6** *Eadmuna paloa* male, Nova Bremen, Santa Catarina, Brazil (image inverted laterally) [CUIC] **7** *Eadmuna paloa* female, Rio Vermelho, Santa Catarina, Brazil (image inverted laterally) [AMNH] **8** *Eadmuna pulverula* holotype female, São Paulo, Brazil [USNM]. Scale bar = 1 cm.

**Figures 9–14.**
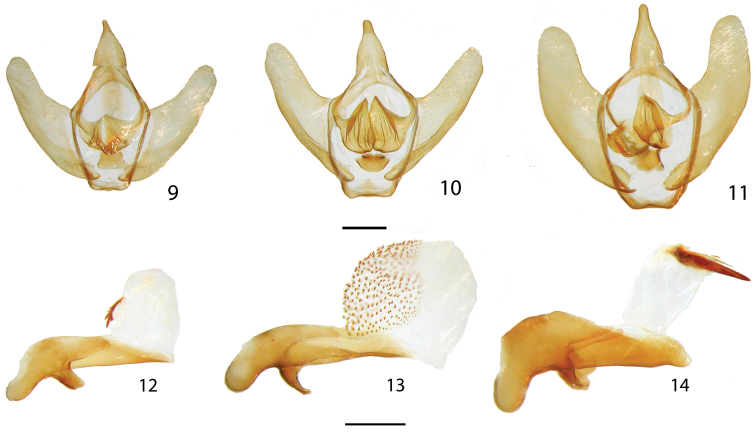
*Eadmuna* male genitalia, valves with phallus and juxta removed, and separated phallus and juxta. **9** *Eadmuna guianensis*, holotype, Mana River, French Guiana [St Laurent diss.: 9-14-14:3] **10** *Eadmuna esperans*, Est. Biol. Boraceia, 850 m, near Salesopolis, São Paulo, Brazil [St Laurent diss.: 9-14-14:6] **11** *Eadmuna paloa*, Nova Bremen, Santa Catarina, Brazil [St Laurent diss.: 9-14-14:8] **12** *Eadmuna guianensis*, holotype, Mana River, French Guiana [St Laurent diss.: 9-14-14:3] **13** *Eadmuna esperans*, Est. Biol. Boraceia, 850 m, near Salesopolis, São Paulo, Brazil [St Laurent diss.: 9-14-14:6] **14** *Eadmuna paloa*, Nova Bremen, Santa Catarina, Brazil [St Laurent diss.: 9-14-14:8]. Scale bars = 1 mm.

**Figures 15–17.**
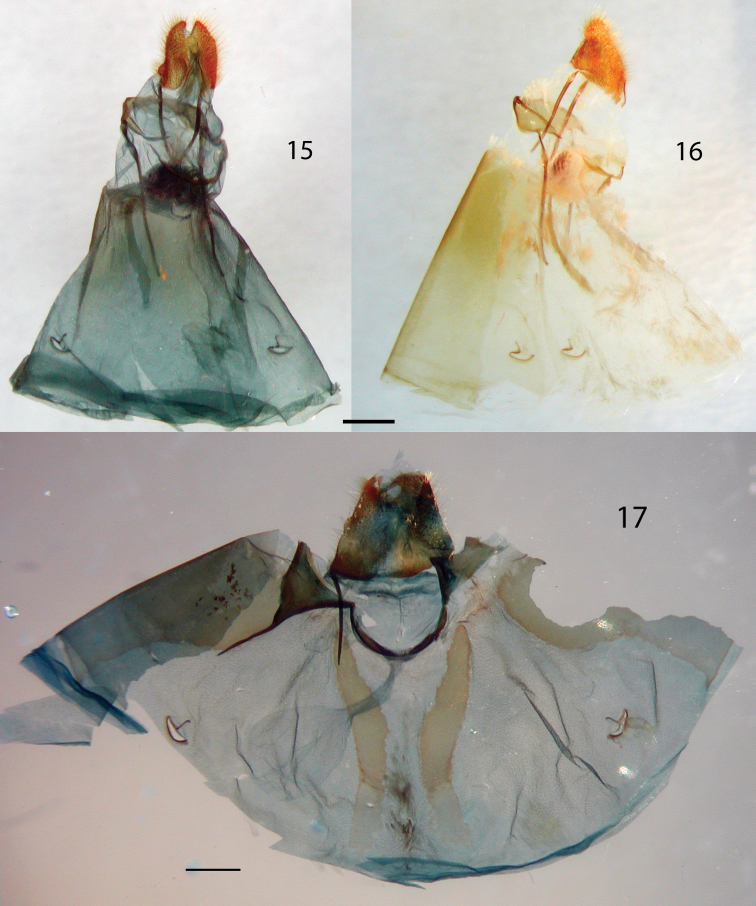
*Eadmuna* female genitalia. **15** *Eadmuna paloa*, ventral, Rio Vermelho, Santa Catarina, Brazil [St Laurent diss.: 10-11-14:3] **16** *Eadmuna paloa*, lateral, Rio Vermelho, Santa Catarina, Brazil [St Laurent diss.: 11-1-14:8] **17** Damaged *Eadmuna pulverula*, holotype, ventral, São Paulo, Brazil [St Laurent diss.: 11-1-14:8]. Scale bars = 1 mm.

**Figure 18.**
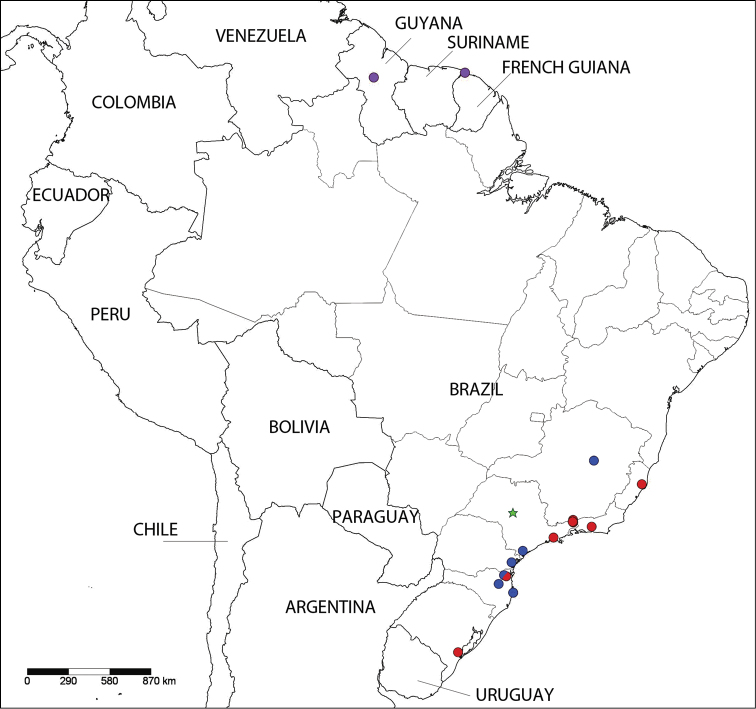
Distribution of *Eadmuna*. *Eadmuna esperans* (red circles), *Eadmuna guianensis* (purple circles), *Eadmuna paloa* (blue circles), *Eadmuna pulverula* (green star). Notes: red/blue circle represents the locality where both *Eadmuna esperans* and *Eadmuna paloa* have been collected, *Eadmuna pulverula* is represented by a star placed near the center of the Brazilian state of São Paulo because the type locality is “São Paulo,” without further information regarding specific locality.

### Eadmuna esperans

Taxon classificationAnimaliaLepidopteraMimallonidae

(Schaus, 1905)

Figs 1 , 2 , 10 , 13 , 18

Cicinnus esperans Schaus, 1905
Eadmuna esperans ; Schaus 1928
Eadmuna esperanza ; Schaus 1933, misspelling
Eadmuna esperans ; Becker 1996

#### Type material.

**Holotype:** BRAZIL, Espírito Santo, Wm. Schaus collection, USNM holotype No.: 8893- genitalia diss: 86062, specimen examined, genitalia preparation not found [♂, USNM]. **Paratypes:** none. Type locality: BRAZIL, Espírito Santo.

#### Additional specimens examined.

All males (21 total): **BRAZIL: Espírito Santo:** Linhares, 40 m, 25–30 i 1998, V.O. Becker coll, Col. Becker No.:113480- St Laurent diss: 11-1-14:9, 11-1-14:10 [4 ♂, USNM]; [no further data]- St Laurent genitalia diss: 11-1-14:6 [1 ♂, USNM]; **Santa Catarina:** “Saint Catherines”- St Laurent genitalia diss: 11-1-14:2 [1 ♂, USNM]; F. Hoffman- St Laurent genitalia diss: 11-1:14:4 [2 ♂, USNM]; Jaraguá [do Sul], 10 x 1934, 14 x 1934, 17 ix 1934, coll. Fritz Hoffmann- St Laurent genitalia diss: 9-14-14:1, Franclemont genitalia slide: number 1763 [3 ♂, CUIC]; **Rio Grande do Sul:** Pelotas 15 v 1953, coll. C.M. de Biezanko no CB: 3503- St Laurent genitalia diss: 9-14-14:4 [1 ♂, CUIC]; **Rio de Janeiro:** Imbariê, 50 m, 21–27 viii 1955, coll. H. Ebert- St Laurent genitalia diss: 9-14-14:5 [1 ♂, CMNH]; “Itatiaya” [Itatiaia], 700 m, 5 x 1928, J.F. Zikan- St Laurent genitalia diss: 11-1-14:3 [1 ♂, USNM]; Zikan- St Laurent genitalia diss: 11-1-14:5 [1 ♂, USNM]; Itatiaia (Maromba), 17 viii 1952, No. 558, Pearson [1 ♂, USNM]; Itatiaia, (L 41, 1300 m) 5/8 iii 1951, Trav. & D. Albuquerque, No. 383- St Laurent diss: 11-1-14:12 [1 ♂, USNM]; PN. Itatiaia, Lago Azul 800(?)m, 12/13 xi 1956, H.R. & G.M. Pearson, No. HRP 1171- St Laurent diss: 11-1-14:13 [1 ♂, USNM]; **São Paulo:** Est. Biol. Boraceia, 850 m, near Salesopolis: 2 x 1971, E.G., I. & E.A. Munroe- St Laurent genitalia diss: 9-14-14:6 [1 ♂, CNC]; 26 ix 1971, E.G., I. & E.A. Munroe [2 ♂, CNC].

#### Diagnosis.

The weakly falcate forewings distinguish *Eadmuna esperans* from the similar *Eadmuna guianensis*, new species, described below. Genitalia of *Eadmuna esperans* are unique among species in the genus in that the vesica has a large scobinate patch as opposed to a single cornutus.

#### Description.

**Male.** *Head*: As for genus but border of darker scales that normally continues down head reaching beneath labial palpi somewhat reduced. *Thorax*: As for genus. *Legs*: As for genus but tibial spurs naked. *Forewing dorsum*: Forewing length: 17–20 mm, avg.: 18 mm, n=19. Triangular, rounded, convex margins becoming concave near subtly accentuated apex. Coloration light silvery brown, suffused with darker brown postmedially except near apex. Hyaline discal spot weakly pronounced, yellowish opaque rather than clear due to covering of yellowish scales, with M_2_ vein separating hyaline patch into two distinct regions. Postmedial line bulging in costal half, scalloped, narrowly interrupted by veins, weaker on costal third except for dark wedge on costa. Occasionally dark diffuse spots between veins immediately beyond center of postmedial line. Antemedial line very weak except for dark chevron on costa. Fringe varies in coloration from darkbrown to off white. *Forewing venter*: As for dorsum but lighter, postmedial line usually much darker, well-pronounced. *Hindwing dorsum*: Rounded, slightly falcate anal angle, bearing similar coloration and pattern as forewing though maculation usually somewhat fainter than on forewing and lacking a hyaline discal spot. *Hindwing venter*: As for dorsum but lighter, postmedial line usually much darker, well pronounced. *Wing venation*: As for genus. *Abdomen*: As for genus, concolorous with thorax. *Genitalia*: n=14. As for genus, simple, but distal end of teardrop-shaped uncus moderately thick, ventral lobes of tegumen subtriangular, with a central sclerotized ridge with three or four secondary ridges ventral to center of subtriangle. Sclerotized plate, dorsal to juxta and phallus, broad, especially on lower half. Phallus, simple, broad, cylindrical, vesica, sac-like with scobinate patch covering roughly half of everted vesica. **Female.** Unknown.

#### Distribution.

This species is known from southeastern and southern Brazil in the states of Espírito Santo (type locality), Rio de Janeiro, São Paulo, Santa Catarina, and Rio Grande do Sul, apparently from relatively low to moderately high elevations (40 to 1300 m). This distribution coincides with both the pampa and Atlantic coastal forest biomes (IBGE 2004).

#### Remarks.

*Eadmuna esperans*, the type species of the genus, was originally described under the genus *Cicinnus*, likely due to the early year of its description, a time when the author, Schaus, was placing many new species in this catch-all genus. It is only known to be sympatric with one congener, *Eadmuna paloa*, in Jaraguá [do Sul], Santa Catarina, Brazil, but can be readily separated by the genital characters mentioned above. *Eadmuna esperans* is most similar to the new species described below, but the latter has more falcate forewings and is known only from the Amazonian region of French Guiana and Guyana. The scobinate patch covering the vesica of *Eadmuna esperans* is unique in the genus.

### Eadmuna guianensis

Taxon classificationAnimaliaLepidopteraMimallonidae

St Laurent & Dombroskie sp. n.

http://zoobank.org/C5715C64-F209-4F6D-9A31-28E01275FDC2

Figs 3 , 4 , 9 , 12 , 18

#### Type material.

**Holotype:** Mana River, Fr. Guiana. May, 1917. Acc. 6008, “*Cicinnus esperans* Schaus,” St Laurent diss.: 9-14-14:3, HOLOTYPE ♂ *Eadmuna guianensis* St Laurent and Dombroskie, 2015 [handwritten red label]. Deposited Carnegie Museum of Natural History. Type locality: French Guiana, Mana River.

**Paratypes:** 4 males: **GUYANA:** 1 male: Tumatumari, Rio Potaro, Br. Guiana, Ac. 5615, St Laurent diss.: 10-27-14:1, PARATYPE ♂ *Eadmuna guianensis* St Laurent and Dombroskie, 2015 [yellow label]. Deposited American Museum of Natural History; **FRENCH GUIANA:** 3 males: Mana River, Fr. Guiana. May, 1917. Acc. 6008, “*Cicinnus esperans* Schaus,” USNM-Mimal: 2510, St Laurent diss.: 11-1-14:1, PARATYPE ♂ *Eadmuna guianensis* St Laurent and Dombroskie, 2015 [yellow label]. Deposited National Museum of Natural History; Mana River, Fr. Guiana. May, 1917. Acc. 6008, PARATYPE ♂ *Eadmuna guianensis* St Laurent and Dombroskie, 2015 [yellow label]. Deposited Carnegie Museum of Natural History; Mana River, Fr. Guiana. May, 1917. Acc. 6008, “*Cicinnus esperans* Schaus,” illegible label, St Laurent diss: 9-14-14:2, PARATYPE ♂ *Eadmuna guianensis* St Laurent and Dombroskie, 2015 [yellow label]. Deposited Cornell University Insect Collection.

#### Diagnosis.

Similar in general appearance to *Eadmuna esperans* but recognizable by darker overall brownish coloration, more acutely, slightly hooked forewing apex, and a vesica bearing a spiked, curved cornutus as opposed to a scobinate patch. The cornutus of *Eadmuna paloa*, unlike that of *Eadmuna guianensis*, is not curved. No other *Eadmuna* is known to occur in northern South America.

#### Description.

**Male.** *Head*: As for genus. *Thorax*: As for genus. *Legs*: As for genus but tibial spurs slightly thinner, half to entirety of spurs covered in fine scales. *Forewing dorsum*: Forewing length: 18–20 mm, avg. 19 mm, n=5. Triangular, convex margins becoming concave near apex, apex accentuated. Brown coloration more predominant than silvery gray, especially distally from thorax, with less extensive speckling due to relative lack of dark, petiolate scales. Discal spot not prominent, elongated, hyaline, yellowish opaque, with M_2_ vein separating hyaline patch into two distinct regions. Postmedial line bulging in costal half, scalloped, narrowly interrupted by veins, weaker on costal third except for dark wedge on costa. Antemedial line weak with dark chevron at costal margin. *Forewing venter*: Darker and lighter areas more finely defined though not particularly darker or lighter overall from dorsum. Postmedial line only somewhat slightly better defined than on dorsum. *Hindwing dorsum*: Rounded, with slightly pronounced anal angle, bearing similar coloration as forewings; postmedial line present, usually well developed, roughly parallel to outer margin, though angled slightly more inward on inner half than in other species. No hyaline patches present. *Hindwing venter*: Darker and lighter areas more finely defined though not particularly darker or lighter overall from dorsum. Postmedial line only somewhat better defined than on dorsum. *Wing venation*: As for genus but R_4_ + R_5_ slightly longer stalked. *Abdomen*: Coloration as for thorax, mostly concolorous with dorsal wing color. *Genitalia*: n=4. As for genus, simple but most structures thinner and weaker than other species. Uncus teardrop shaped, extended apically, very thin apically, highly truncated basally. Ventral lobes of tegumen subtriangular, ridged; ridges thicker and more pronounced than for *Eadmuna esperans*. Valves simple, relatively thin. Sclerotized plate, dorsal to juxta and phallus, broad, rounded dorsally. Phallus, simple, cylindrical, pointed when viewed ventrally/dorsally, vesica sac-like, bulbous with single curved cornutus bearing four or five spikes that increase in size distally. **Female.** Unknown.

#### Etymology.

Named for the Guianas from where all the specimens were collected.

#### Distribution.

This species is only known from Guyana and French Guiana and thus represents a significant disjunction in geographic distribution of the genus, the other three *Eadmuna* species being found in southern and southeastern Brazil.

#### Remarks.

*Eadmuna guianensis* is known from the Amazon Rainforest, very distant from the range of its three congeners. This disjunction is unlikely to be due to an artefact of under-sampling in intervening areas because the Amazon region is well collected for Mimallonidae (R. St Laurent pers. obs.). Despite the seemingly geographic isolation and distance from the localities of the other species of *Eadmuna*, this species clearly belongs to this genus due to the characters of the genitalia, which are very similar to those of the type species and, surprisingly, bear aspects similar to *Eadmuna esperans* in wing pattern and valve structure and to *Eadmuna paloa* in the vesica.

### Eadmuna paloa

Taxon classificationAnimaliaLepidopteraMimallonidae

Schaus, 1933 rev. status

Figs 5–7 , 11 , 14 , 15–16 , 18

Eadmuna esperans ; Becker 1996, incorrect synonymy

#### Type material.

**Holotype:** BRAZIL, São Paulo, “No. 71,” USNM holotype No.: 34362- St Laurent diss: 11-1-14:7 [examined] [♂, USNM]. **Paratypes:** none. Type locality: BRAZIL, São Paulo.

#### Additional specimens examined.

22 males, 2 females: **BRAZIL: São Paulo:** Jacupiranga, 800m, 8 ii 1993, V.O. Becker, Col. Becker no. 87164- St Laurent diss: 11-1-14:11 [1 ♂, USNM]; **Santa Catarina:** Rio Vermelho: i 1957, A. Maller col., No. 1714 [1 ♂, USNM]; ii 1945, i 1944, leg. Anton Maller- St Laurent diss: 10-11-14:2 [2 ♂, AMNH]; ii 1945- leg Anton Maller- St Laurent diss: 10-11-14:3 [1 ♀, AMNH]; ii 1944, A. Maller Coll., Frank Johnson Donor- St Laurent diss: 11-12-14:1 [1 ♀, AMNH]; Hansa Humbolt [Corupá] [probably pre 1944] [1 ♂, USNM]; Jaraguá [do Sul], 29 xi 1934, 17 ix 1934, coll. Fritz Hoffmann- St Laurent genitalia diss: 9-14-14:7, Franclemont genitalia diss: 1769 [2 ♂, CUIC]; Nova Bremen, 7 xii 1936, 14 x 1936, 18 v 1936, 7 ix 1935 coll. Fritz Hoffmann- St Laurent genitalia diss: 9-14-14:8 [4 ♂, CUIC]; [no further data] [3 ♂, USNM]; F. Hoffman, No. 13791 [1 ♂, USNM]; F. Hoffman [1 ♂, USNM]; **Paraná:** Banhados (RR. from Curitiba to Paranaguá), 800 m, 14 ii 1972, E.G., I. & E.A. Munroe- St Laurent diss: 10-5-14-:1 [2 ♂, CNC]; **Minas Gerais:** Diamantina, Serrinha- X-IV, with X-IV crossed out, leg. E. Cohn- St Laurent diss: 10-11-14:1 [4 ♂, AMNH].

#### Diagnosis.

*Eadmuna paloa* has more elongate forewings with larger hyaline areas than any other *Eadmuna* species. The vesica has a single, large, straight cornutus that is fused to progressively smaller, parallel cornuti that transition into a mane of long, clear, hair-like projections that originate from the vesica. Additionally, the lobes of the basal half of the tegumen are much more heavily sclerotized in all *Eadmuna paloa* examined than in other species in the genus. The female is larger than the male, with broader wings and darker, more pronounced antemedial and postmedial lines. The female of *Eadmuna paloa* is similar to the female of *Eadmuna pulverula*, but the forewings are less falcate, with larger hyaline patches, and there is no longitudinal dark line on the venter of the abdomen.

The primary genital characters used to differentiate *Eadmuna esperans* and *Eadmuna paloa* are the vesica and cornutus. In *Eadmuna esperans* the vesica is sac-like and covered in a scobinate patch whereas the vesica of *Eadmuna paloa* is thinner and more cylindrical, and bears a single large cornutus. Aside from the very good genitalia characters, the two species can also be readily differentiated by wing morphology. *Eadmuna paloa* is generally more silvery in color with more acutely triangular forewings, has much larger forewing hyaline areas, and males have less pronounced postmedial lines.

#### Description.

**Male.** *Head*: As for genus, but more off white in color rather than straw colored; dorsal surface of labial palpi and area surrounding eyes covered in contrasting brown scales. Labial palpi and antennal tufts smaller. *Thorax*: As for genus, but as on head, scales of thorax lighter in coloration than in other species, thus darker petiolate scales more pronounced. *Legs*: As for genus, but tibial spurs clothed in small scales varying from covering proximal half to near entirety. *Forewing dorsum*: Forewing length: 16–20mm, avg. 18 mm, n=16. As for genus, but more acutely triangular, convex margins not concave near apex, lower quarter of forewing bows out slightly. Silvery gray brown with especially contrasting, extensive speckling due to dark, petiolate scales. Postmedial region roughly concolorous with rest of forewing, though silvery sheen lost near margin, so margin a singed-brown color. Hyaline discal spot prominent, large, very clear, not covered in scales, outlined by dark scales, M_2_ separates hyaline patch into two distinct regions, creating a rough B-shape. Very faint postmedial line bulging in costal half, dentate, narrowly interrupted by veins, weaker on costal third except for darker wedge on costa. Antemedial line faint. Fringe white, contrasting with darker brown edge of wing. *Forewing venter*: As for dorsum, but lighter overall; postmedial line usually much darker. *Hindwing dorsum*: Rounded, slightly pronounced anal angle, bearing similar coloration to forewings. Postmedial line, when present, may be more strongly marked than on forewing. No hyaline patches present. Fringe as for forewing. *Hindwing venter*: As for dorsum, but lighter, postmedial line usually much darker. *Wing venation*: As for genus. *Abdomen*: As for genus, concolorous with thorax, but silvery instead of straw-colored. *Genitalia*: n=8. As for genus, uncus simple, teardrop shaped, extended apically with moderate thickness distally. Ridged ventral lobes of tegumen subtriangular, prominently sclerotized. Ridges thinner than for other species, and thus sharper and flatter, with central ridge especially pronounced. Valves simple, short and stocky for genus, bent upwards at a roughly ninety degree angle so distal ends of valves more in parallel with uncus than angled away. Sclerotized plate, dorsal to juxta and phallus, truncated dorsally with two heavily sclerotized points. Phallus, simple, cylindrical, distal end rounded, vesica elongated with single large cornutus fused to progressively smaller parallel cornuti transitioning into a mane of long, clear, hair-like projections that originate from vesica near base of cornutus, reaching outwards to surround cornutus. **Female.** *Head*: As for male, antennae bipectinate. *Thorax*: As for male. *Legs*: As for male, but small scales nearly completely cover tibial spurs. *Forewing dorsum*: Forewing length: 22–24 mm, avg. 23 mm n=2. As for male but much broader. Postmedial region lighter, more silvery-grey than medial area. Hyaline discal mark large, prominent. Postmedial line, more pronounced than for male, brown, dentate, narrowly interrupted by veins, dark wedge where postmedial line meets costa. Antemedial lines, bilobed, B-shaped. *Forewing venter*: As for dorsum, but lighter, postmedial line more contrasting. *Hindwing dorsum*: As for male, but broader, with hardly accentuated anal angle, essentially bearing same coloration as forewing. Unlike in male, entire hindwing, save for postmedial line, concolorous silvery gray, without a brown edge and without darker medial area on forewing. Dentate postmedial line dark and well pronounced, narrowly interrupted by veins, slightly darker than forewing ground color. No hyaline patches present. *Hindwing venter*: As for dorsum, but lighter, postmedial line more contrasting. *Wing venation*: As for genus. *Abdomen*: Much thicker than that of male. Color as for thorax, though darkening somewhat distally. *Genitalia*: n=2. Papillae anales elongated, covered in fine setae, apophyses posteriores about half length of apophyses anteriores, so that when abdominal segments fully distended apophyses posteriores extend about to posterior margin of eighth segment. Ductus bursae short, ostium opening immediately into corpus bursae. Corpus bursae firm, round, with heavily-sclerotized, internal bar-like structures reinforcing membrane, appendix bursae elongated. Two very elongated, thin sclerotized plates on venter of eighth segment.

#### Distribution.

This species is known only from southeastern and southern Brazil. São Paulo is the type locality, which was erroneously reported as Paraguay in Becker (1996). In southern Brazil, specimens were examined from the states of Santa Catarina and Paraná. *Eadmuna paloa* is also known from Diamantina, Minas Gerais from four specimens in the AMNH. This record is of considerable distance from the other localities closer to the coast and falls within the Cerrado biome (IBGE 2004). The only elevation data comes from the two Paraná specimens and the Jacupiranga, São Paulo locality, which are of moderate elevation, both localities sited at 800 m.

#### Remarks.

*Eadmuna paloa* was synonymized with *Eadmuna esperans* by Becker (1996) without justification. The genitalia of the two species are shown to be substantially different, particularly the vesica and presence/absence of a cornutus. Both species are found to be sympatric, at least in Jaraguá [do Sul], Santa Catarina, Brazil.

This work describes the first female specimens to be attributed to *Eadmuna*. The two female specimens from the AMNH are part of a series of *Eadmuna paloa* from Rio Vermelho, Santa Catarina, Brazil, which includes two male specimens that, based on wing morphology and genitalia characteristics, match the male holotype of *Eadmuna paloa* from São Paulo. The wing morphology of the females is very similar to that of the males, particularly the silvery-gray coloration, highly dentate postmedial lines on all wings, and the presence of a large hyaline patch on the forewing. Additional support for associating these females with *Eadmuna paloa* is that the corpus bursae is highly sclerotized and strongly reinforced, potentially protecting the more membranous material of the corpus bursae from puncture due to the highly sclerotized and very sharp cornutus of the male (B. C. Schmidt pers. comm.). Males of *Eadmuna esperans* do not bear cornuti, only a scobinate patch on the vesica, thus relatively reduced sclerotization of the corpus would be expected in the female of *Eadmuna esperans*. The two females from Santa Catarina are therefore most reasonably associated with *Eadmuna paloa* males, which are much more frequent in collections.

### Eadmuna pulverula

Taxon classificationAnimaliaLepidopteraMimallonidae

(Schaus, 1896) comb. n.

Figs 8 , 17 , 18

Perophora pulverula Schaus, 1896
Cicinnus pulverula ; Schaus 1928
Cicinnus pulverula ; Becker 1996

#### Type material.

**Holotype:** BRAZIL, São Paulo, Wm. Schaus collection, USNM holotype No.:12563- St Laurent diss: 11-1-14:8 [examined] [♀, USNM]. **Paratypes:** none. Type locality: BRAZIL, São Paulo.

#### Diagnosis.

Similar to female of *Eadmuna paloa* but the forewing apex is more falcate, the forewing discal hyaline patch slightly smaller, and with a distinct, thin dark line along the venter of the abdomen from the thorax to the distal end.

The papillae anales in *Eadmuna pulverula* are much broader and stockier than in *Eadmuna paloa*, the apophyses anteriores and posteriores are approximately the same length in *Eadmuna pulverula* whereas the apophyses posteriores are shorter than the apophyses anteriores in *Eadmuna paloa*. Sclerotized, ribbon-like plates are located on the venter of the eighth abdominal segments in both species, but those of *Eadmuna pulverula* are wider and angled inward toward each other medially, but are more parallel in *Eadmuna paloa*. Finally, the corpus bursae of *Eadmuna pulverula* lacks any sclerotized structure, but in *Eadmuna paloa*, this is the most distinctive trait of the genitalia.

#### Description.

**Female.** *Head*: Antennae bipectinate. *Thorax*: As for female of *Eadmuna paloa*. *Legs*: As for female of *Eadmuna paloa*, but small scales nearly completely cover tibial spurs. *Forewing dorsum*: Forewing length: 24 mm, n=1. As for female of *Eadmuna paloa* but with slightly more pronounced apex and overall darker coloration and heavier speckling due to higher number of petiolate scales. Hyaline discal mark smaller. Postmedial line present, darker, thicker, brown, dentate, narrowly interrupted by veins, dark wedge where postmedial line meets costa. Antemedial lines present, bilobed, B-shaped, but straighter. *Forewing venter*: As for dorsum, postmedial line more contrasting. *Hindwing dorsum*: Coloration as for forewing though lighter overall, anal angle accentuated. Postmedial line dentate, dark, well pronounced, narrowly interrupted by veins, slightly lighter than that of forewing. No hyaline patches present. *Hindwing venter*: As for dorsum, but lighter, especially in antemedial area. *Wing venation*: As for genus. *Abdomen*: Very robust, color similar to that of thorax, though yellowing somewhat in holotype, likely due to age of specimen. Longitudinal dark line along middle of abdominal venter formed by darkbrown, thin, petiolate scales. *Genitalia*: n=1. Papillae anales stocky, somewhat triangular, covered in fine setae, apophyses posteriors and anteriores of similar length, though apophyses posteriors slightly thicker, only one of each apophysis present in holotype specimen due to damage. Ductus bursae short, corpus bursae small, baglike, without signum or cornuti. Remnants of appendix bursae visible. Wide, elongated, sclerotized plates present of venter of eighth segment, curving inward toward each other, roughly midway along their length. **Male.** Unknown.

#### Distribution.

Known only from the type specimen, collected in São Paulo; no further locality information is available. Distribution is represented in Fig. 18 by a green placeholder star near the center of the state of São Paulo; however, it may be inferred from the distributions of *Eadmuna esperans* and *Eadmuna paloa* that *Eadmuna pulverula* likely ranges farther to the east in the state of São Paulo nearer to the coastal Atlantic Forest.

#### Remarks.

The holotype of *Perophora pulverula* was determined to be a female of an *Eadmuna* species due to its close similarity to female *Eadmuna paloa* from Santa Catarina, Brazil. Despite the fact that female *Eadmuna* had not been recognized prior to this work, it can be reasonably determined that the females from Santa Catarina are in fact *Eadmuna paloa* (see remarks of *Eadmuna paloa*) whereas the female of *Eadmuna pulverula* most likely represent a distinct species based on differences in genitalia.

Unfortunately, the genitalia of the holotype of *Eadmuna pulverula* are not intact (see Fig. 17) and thus are not entirely available for study. However, the genitalia characters that are present are very distinct from either of the Santa Catarina *Eadmuna paloa* females, which were both similar to each other. The size differences between the two taxa are among the most striking. Although the overall size of the females of *Eadmuna paloa* and *Eadmuna pulverula* are very similar, the genitalia of *Eadmuna pulverula* are nearly twice as large as those of *Eadmuna paloa* in all respects.

It is possible that *Eadmuna pulverula* is the unidentified female of *Eadmuna esperans* due to process of elimination in that the only *Eadmuna* known to occur in southern and southeastern Brazil are *Eadmuna paloa* and *Eadmuna esperans* and the female of *Eadmuna paloa* has been identified. However, there is not enough evidence to support *Eadmuna pulverula* and *Eadmuna esperans* as being conspecific. A major problem with considering *Eadmuna pulverula* to be the female of *Eadmuna esperans* is the wing color. Females of *Eadmuna paloa* are so similar to conspecific males that one would expect the female of *Eadmuna esperans* also to be very similar to conspecific males, and not exhibit the extreme dimorphism that would be present if *Eadmuna pulverula* was considered conspecific with *Eadmuna esperans*. Extreme sexual dimorphism in wing color and pattern is not common in Mimallonidae, aside from the fact that females are usually larger than males, with much broader wings (R. A. St Laurent pers. obs.). In actuality, *Eadmuna pulverula* is very similar to female *Eadmuna paloa*, with major differences only in the genitalia.

The genitalia of *Eadmuna pulverula* are so distinct from the females of *Eadmuna paloa* that it becomes impossible to consider them the same entity which, based on wing morphology alone, would have been the most logical conclusion pending further evidence. The most conservative approach in dealing with the name *Eadmuna pulverula* is to transfer it to *Eadmuna* from *Cicinnus* due to the female holotype bearing a striking similarity to female *Eadmuna paloa*, but to maintain it as a valid species rather than trying to associate it with cryptic males currently considered *Eadmuna paloa* or attributing it to *Eadmuna esperans* by mere process of elimination. Until female *Eadmuna esperans* are accurately associated with the easily recognizable males, the current placement of *Eadmuna pulverula* remains somewhat inconclusive.

## Supplementary Material

XML Treatment for Eadmuna

XML Treatment for Eadmuna esperans

XML Treatment for Eadmuna guianensis

XML Treatment for Eadmuna paloa

XML Treatment for Eadmuna pulverula
